# Anticancer potential of *Phoenix dactylifera* L. seed extract in human cancer cells and pro-apoptotic effects mediated through caspase-3 dependent pathway in human breast cancer MDA-MB-231 cells: an in vitro and in silico investigation

**DOI:** 10.1186/s12906-022-03533-0

**Published:** 2022-03-15

**Authors:** Mohsin Ali Khan, Romila Singh, Sahabjada Siddiqui, Imran Ahmad, Rumana Ahmad, Shivbrat Upadhyay, Md. Abul Barkat, Ahmed Mahmoud Abdelhaleem Ali, Qamar Zia, Aditi Srivastava, Anchal Trivedi, Ishrat Husain, Anand Narain Srivastava, Durga Prasad Mishra

**Affiliations:** 1 Research and Development Unit, Era’s Lucknow Medical College and Hospital, Era University, Lucknow, 226003 India; 2 Cell Death Research Laboratory, LSS-106, Endocrinology Division, CSIR-Central Drug Research Institute, Jankipuram Extension, Lucknow, 226031 India; 3 Department of Biotechnology, Era’s Lucknow Medical College and Hospital, Era University, Lucknow, 226003 India; 4 Department of Biochemistry, King George’s Medical University, Lucknow, 226003 India; 5 Department of Biochemistry, Era’s Lucknow Medical College and Hospital, Era University, Lucknow, 226003 India; 6 Department of Pharmaceutics, College of Pharmacy, University of Hafr Al-Batin, Al Jamiah, Hafr Al Batin, 39524 Saudi Arabia; 7 Department of Pharmaceutics and Industrial Pharmacy, College of Pharmacy, Taif University, P. O. Box 11099, Taif, 21944 Saudi Arabia; 8 Health and Basic Science Research Centre, Majmaah University, Majmaah, 11952 Saudi Arabia; 9 Department of Medical Laboratory Sciences, College of Applied Medical Sciences, Majmaah University, Majmaah, 11952 Saudi Arabia; 10 Department of Pathology, Era’s Lucknow Medical College and Hospital, Era University, Lucknow, 226003 India

**Keywords:** *Phoenix dactylifera* seed extract, Phytoconstituents, Human breast cancer, Cytotoxicity, HPLC, Molecular docking

## Abstract

**Background:**

*Phoenix dactylifera* L. has a diverse set of pharmacological properties due to its distinct phytochemical profile. The purpose of this study was to investigate the anticancer potential of *Phoenix dactylifera* seed extract (PDSE) in human breast cancer MDA-MB-231 and MCF-7 cells, as well as liver cancer HepG2 cells, and to investigate the anticancer efficacy in triple-negative MDA-MB-231 cells, followed by in silico validation of the molecular interaction between active components of PDSE and caspase-3, an apoptosis executioner protein .

**Methods:**

In this study, human cancer cell lines were cultured and subsequently treated with 10 to 100 μg/mL of PDSE. MTT test was performed to determine the cell viability, MMP was measured using fluorescent probe JC-1, nuclear condensation was determined by Hoechst 33258 dye, Annexin V-FITC & PI staining and cell cycle analysis were evaluated through flow cytometer, and apoptotic markers were detected using western blotting. The bioactive agents in PDSE were identified using high-performance liquid chromatography (HPLC) analysis. The binding affinity was validated using molecular docking tools AutoDock Vina and iGEMDOCK v2.1.

**Results:**

Cell viability data indicated that PDSE inhibited cell proliferation in both breast cancer cells and liver cancer cells. MDA-MB-231 cells showed maximum growth inhibition with an IC_50_ value of 85.86 μg/mL for PDSE. However, PDSE did not show any significant toxicity against the normal Vero cell line. PDSE induced MMP loss and formation of apoptotic bodies, enhanced late apoptosis at high doses and arrested cells in the S phase of cell cycle. PDSE activated the enzymatic activity of cleaved caspase-3 and caused the cleavage of poly-ADB ribose polymerase (PARP) protein. PDSE upregulated pro-apoptotic Bax protein markedly but  no significant effect on tumor suppressor protein p53, while it downregulated the anti-apoptotic Bcl-2 protein expression. HPLC analysis showed the presence of rutin and quercetin bioactive flavonols in ethanolic extract of PDS. Interestingly, both active components revealed a strong binding interaction with amino acid residues of caspase-3 (PDB ID: 2XYP; Hetero 4-mer - A2B2) protein.

**Conclusion:**

PDS could serve as a potential medicinal source for apoptotic cell death in human breast cancer cells and, thus, could be used as a promising and crucial candidate in anticancer drug development. This study warrants further in vivo research, followed by clinical investigation.

**Supplementary Information:**

The online version contains supplementary material available at 10.1186/s12906-022-03533-0.

## Background

Cancer is an ensemble of diseases that develop over a long stretch of time, causing millions of mortalities across the globe [1]. Owing to its extremely aggressive nature and poor prognosis and survival rate, cancer remains an important public health issue worldwide. Amongst various cancers, breast and liver cancer are of great concern globally, accounting for 2.26 and 2.21 million new cases, respectively in 2020 [2]. Recent cancer statistics have suggested a significant rise in the number of breast cancer patients, indicating breast cancer (11.7%), as a most prevalent type of cancer diagnosed every year, followed by lung (11.4%), colorectal (10.0%), prostate (7.3%), and stomach (5.6%) cancers [3]. As per the statistic, 1 out of 4 cancer patients are diagnosed with breast cancer, causing 1 out of 6 cancer related mortalities [4]. Among Indian females, breast cancer ranks as number one cancer, having a mortality rate of 12.7 per 100,000 women [5]. The incidence of liver and breast cancers has increased in many parts of the world, notably in India, North and South America, as well as in most European countries.

Natural products have emerged as a benchmark in the process of discovery and development of novel drugs, particularly for anticancer and anti-infectious agents [6]. It is noteworthy that about fifty percent of anti-cancer drugs and therapies are either derived from natural sources or natural products [7]. A range of herbal derived components such as alkaloids, polyphenols, flavonoids, terpenoids and polysaccharides are being used against various cancers and communicable diseases [8, 9]. Various therapeutic strategies and interventions are available for these diseases; however, most of them remain incurable because of drug resistance. In addition, some natural products are not free from side effects. Therefore, the search and discovery of novel plant-derived products are essential to improve the impact and outcome of plant-based therapeutic agents.

Ajwa dates are the fruits of the native tree *Phoenix dactylifera* L. of Saudi Arabia. For thousands of years in Egypt and the Middle East, the tree has been enlisted for its diverse medicinal uses in Pharmacopeia [10]. All parts of the plant, particularly fruits, seeds, leaves, flowers and roots, are utilized in Ayurveda-based formulations and have a significant role as a Rasayana *i.e.* rejuvenation medicine [11]. The therapeutic effects of date palm have been well-documented [12]. Ajwa dates are cultivated only in Saudi Arabia/Al-Madinah Al-Munawara and be effective against several types of diseases [13]. Ajwa date seeds have been utilized in Egyptian traditional medicine to treat a variety of infectious disorders diabetes and cancer [14, 15]. Traditionally and historically, Ajwa dates are used mainly for their antioxidant, anti-inflammatory and hepatoprotective, anticancer and cardiac function improvement effect [16, 17].

Primary breast cancer is confined to the milk glands or ducts of the breast. However, in some cases, primary breast cancer becomes aggressive and spreads to distant organs such as the liver causing metastasis [18]. Therefore, in the present study, cancer cell lines from two different tissues *viz.* breast (MDA-MB-231 and MCF-7), and liver (HepG2) have been used to investigate the anticancer potential of *Phoenix dactylifera* seed extract (PDSE). African green monkey normal kidney epithelial cell line (Vero) has been widely used in various toxicological, virology and pharmacological research domains, particularly as a sensitive model for toxicity assays of compounds of various nature, either chemical or microbial toxins [19–22]. Thus, to test the toxicity of PDSE, Vero cell line was used as a control to mimic normal mammalian cells. HPLC characterization was carried out to detect the presence of active phytoconstituents and rutin and quercetin were found in the extract. In silico computational analyses revealed that these active ingredients of PDSE displayed excellent binding interaction with caspase-3 protein. Thus, the present study indicates that PDSE might be utilized as an adjunct to the mainline of cancer treatment or be developed as a therapeutic anti-cancer agent in breast cancer therapy.

## Methods

### Reagents and chemicals

DMEM/F-12 media, fetal bovine serum, penicillin and streptomycin solution, rutin as well as quercetin flavonoids were procured from Sigma Aldrich, USA. Hoechst 33342 dye and MTT were purchased from Himedia, India. All the reagents utilized were of analytical grade.

### PDSE preparation

Fresh Ajwa dates were obtained from a farmer of Al- Madina Al- Munawwarah city in the Kingdom of Saudi Arabia with the permission of the landowner (farmer). Mr. Muhammad Arif, Asst. Prof., Pharmacognosy Department, Integral University, Lucknow (specimen no. IU/PHAR/HRB/14/21) identified the plant material. The use of seed material in this study complied with international, national and institutional guidelines. The ethanolic extract of plant material was prepared as reported earlier with minor modifications [23]. Date fruit seeds were manually separated, washed with double distilled water, oven-dried, and coarsely powdered with a pestle and mortar. The coarse powder content was then extracted in 95% ethanol (1:3 w/v ratios) at 25 °C for three days. The extracted solvents were collected and filtered using Whatman No. 1 filter paper (125 mm). Rotavapor (BuchiRotavapor R-205, Switzerland) was used to concentrate the filtrate in a vacuum at 45 °C. The extract was concentrated further in a water bath until it formed a semi-solid paste. The extract was then stored at room temperature in an airtight container for two weeks. After getting the powdered form of PDSE, it was stored in the refrigerator at 4 ^o^C until it was used in tests.

### HPLC analysis of PDSE

 An Waters 515 HPLC Pump system (Milford, USA) equipped with a W2998 PDA detector, a pump control module, a Waters column temperature controller, and an empower chromatography workstation was utilized to characterize PDSE, as reported previously [23]. An X BRIDGE C18 5 m, 4.6 × 250 mm reverse-phase column with gradient elution as the mobile phase was used for chromatographic analysis. The mobile phase was a gradient of water (Solvent A) and acetonitrile (Solvent B). Rutin and quercetin were used as standards. HPLC was examined at 257 nm to obtain real-time chromatograms of both standards and PDSE.

### Cell line and culture

Human triple-negative MDA-MB-231 breast cancer, human ER and PR positive MCF-7 breast cancer, human liver HepG2 carcinoma , and normal kidney epithelial Vero cell lines were purchased from the NCCS, Pune, India. Cells were cultured in DMEM:F12 (1:1) medium in 25 cc tissue culture flasks in an incubator (Thermo Scientific, USA) as reported previously [24]. 

### MTT assay

The anti-proliferative activity of PDSE was done by MTT assay using an established protocol [25]. Briefly, all three cancer cell lines *viz.* MDA-MB-231, MCF-7, HepG2 and one normal kidney epithelial cell line Vero were seeded at a density of 1 × 10^4^ cells/mL in 96-well microtiter culture plates overnight. PDSE was dissolved in culture media for stock preparation and diluted in the same media at various concentrations ranging from 10, 25, 50, 75, and 100 μg/mL for treatment of cultured cells over a 24- and 48-h period. At the end of the incubation period, absorbance values were read using MTT dye (5 mg/mL stock) through an ELISA plate reader (Biorad-PW41, USA) at 550 nm. IC_50_ values were calculated using GraphPad Prism software. 

### Nuclear condensation assay

The apoptosis-inducing effect of PDSE was assessed at two effective doses of 50 and 100 μg/mL. Hoechst 33258 staining was used to assess nuclear condensation, as described previously [23]. In brief, cells fixation was performed using 4% paraformaldehyde following exposure with 50 and 100 µg/mL of PDSE for 48 h. After permeabilization and then staining with Hoechst 33258 (5 μg/mL) dye, cells were photographed under an inverted fluorescence microscope (Zeiss AxioVert 135, US).

### Measurement of intracellular ROS 

Flow cytometry technique was used to assess the intracellular ROS levels using DCFH-DA dye as described previously [23]. After 12 h exposure of PDSE,  cell were washed with PBS and incubated in PBS containing 10 μM DCFH-DA dye at 37 °C for 20 min. The cells were then washed twice with PBS and subjected to flow cytometry analysis (FACS Canto II flow cytometer, BD Biosciences, USA).

###  Evaluation of mitochondrial membrane potential (MMP, ΔΨ_m_)

The fluorescent probe JC-1 was used to assess MMP changes as reported previously [23]. Briefly, cells were treated with 50 and 100 μg/mL of PDSE for 48 h. For flow cytometry analysis, cells were incubated with JC-1 dye at a final concentration of 2 μM for 30 min in the dark. After washing with PBS twice, cells were resuspended in 500 μL PBS and analyzed using flow cytometry.

### Determination of apoptosis by Annexin V-FITC & PI double stain

Flow cytometry was used to quantify apoptotic cells using an Annexin V-FITC Apoptosis Kit (BioVision, USA) manufacturer’s protocol. In brief, cells at a density of 1 × 10^6^ cells/mL were incubated for 48 h with PDSE at concentrations of 50 and 100 μg/mL. Cells were then harvested, resuspended in binding buffer and stained for 15 min at 25 °C in the dark with 2 μL Annexin V-FITC and 2 μL PI. Flow cytometry was used to assess the apoptotic index.

### Analysis of cellular DNA content

Cells at density 1 × 10^6^ cells/mL were used to  treat with 50 and 100 μg/mL concentrations of PDSE into a 6-well plate for 48 h. Flow cytometry was used to examine different stages of the cell cycle and the contents of cellular DNA, as described previously [26].

### Western blot analysis

The western blotting of PDSE treated and untreated cells was carried out as per a previously published method [27]. Briefly, cell lysates were prepared in ice-cold RIPA lysis buffer containing protease and phosphatase inhibitor cocktail (Thermo Scientific). Protein sample (30 μg each) was resolved on a 10-15% SDS-PAGE gel, transferred to the nitrocellulose membrane (Millipore). Finally, the immunodetection was done using enhanced chemiluminescence (Millipore) as per manufacturer’s instructions. The full-length blots were cut before antibody hybridization and each section was incubated with primary antibody individually. Image J software (version 1.43, NIH, USA) was used to quantify the relative abundance of each band against housekeeping β-actin protein.

### Molecular docking analysis through AutoDock Vina and iGEMDOCK v2.1

The binding interaction(s) of PDSE active components *viz.* rutin and quercetin with the apoptosis executioner protein caspase-3 was performed using AutoDock 4.2 and iGEMDOCK v2.1 [24]. PubChem database was used to access the 3D structures of rutin and quercetin components with PubChem CID: 5280805 and 5280343, respectively. Energy minimization of phytocomponents was done by ChemBio3D Ultra 14.0, with Force Field type MM2. The 3D X-ray diffraction crystal structures of caspase-3 protein (PDB ID: 2XYP; Hetero 4-mer - A2B2) were downloaded from RCSB Protein Data Bank. Complete PDB structure with no mutation and resolution 1.86 Å was selected for molecular docking study. Before docking, the refinement procedure was carried out by the addition of missing atoms to the residues, addition of polar hydrogen atoms and Kollman charges, removal of crystallographic water molecules, and external and irrelevant ligands and ions from the protein. AutoDock Vina reduces the computational effort required for binding pocket predictions. This tool was used to run docking simulations and to generate ten ligand-receptor complex conformations that were then ranked based on binding energy. iGEMDOCK can be used for post-screening analysis and predicting pharmacological interactions from screening compounds. In case of iGEMDOCK v2.1, genetic algorithm parameters were as follows: population size = 200, generations = 0 and number of solutions = 2. The best fitted was then selected displaying total binding energy in the form of van der Waals forces (VDW), hydrogen bond (HB) and electrostatic interactions (EI). AccelrysBiovia Discovery Studio 2017 R2 (Biovia, San Diego, CA, USA) was used to visualize the best docking sites and poses from both docking simulations [24, 28].

### Statistical analysis 

 Cell viability data were expressed as the mean ± SEM of at least three independent experiments. GraphPad Prism software was used for statistical analysis, which comprised one-way ANOVA and Dunnett’s Multiple Comparison Test (Version 5.01). A *p*-value of less than 0.05 was determined statistically significant.

## Results

### HPLC characterization of PDSE

**Fig. 1:**
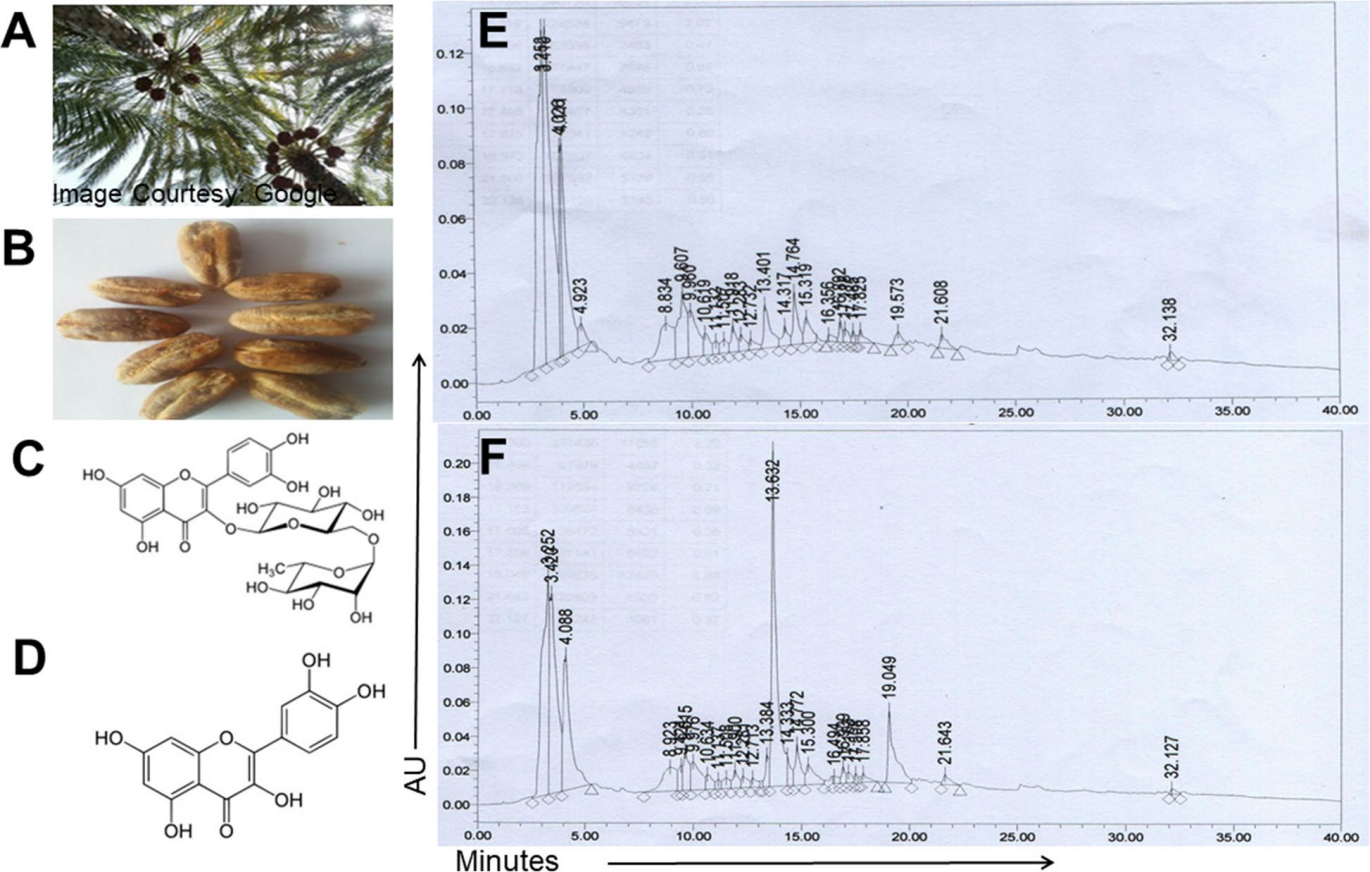
HPLC profile of standards rutin and quercetin and PDSE. **a** and **b** Photographs of Ajwa date plant and fresh Ajwa seeds, respectively **c** and **d** Chemical structures of rutin and quercetin **e** HPLC chromatogram of PDSE **f** HPLC chromatogram of rutin (R_t_ = 13.401 min) and quercetin (R_t_ = 19.573 min) along with PDSE

Figure 1a and b show photographs of Ajwa date plant and fresh Ajwa seed, respectively, whereas Fig. 1c and d show the chemical structure of the active components rutin and quercetin, respectively. Figure 1e represents the chromatogram of PDSE while Fig. 1f represents the chromatogram of reference standards rutin and quercetin along with PDSE. The peak area and percentages of different components with a specific retention time (R_t_) in HPLC chromatograms are shown in Table S1. The HPLC chromatographic analysis provided a fine separation of rutin and quercetin with R_t_ value of 13.632 and 19.049 min, respectively at 257 nm in chromatograms (Fig. 1f). The corresponding peak of rutin and quercetin in PDSE was found with R_t_ of 13.401 and 19.573 min, respectively under similar conditions (Fig. 1e). This study revealed the presence of rutin and quercetin as active components in PDSE.

### Effect of PDSE on cellular morphology and cell viability 

**Fig. 2:**
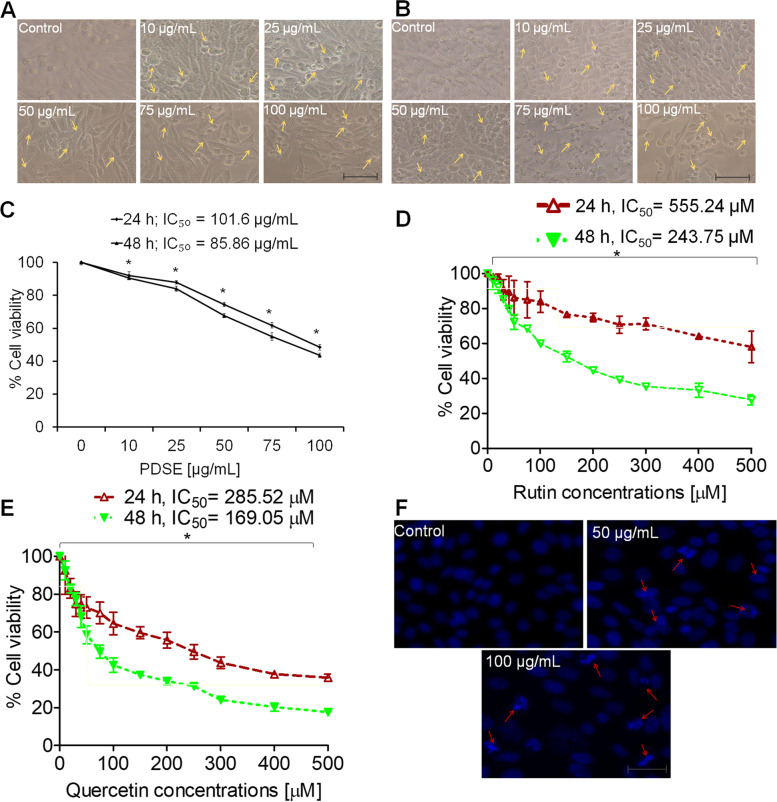
Evaluation of the cytotoxic activity of PDSE against human MDA-MB-231 cells at different concentrations (10–100 μg/mL) using phase contrast microscope. **a** and **b** Photomicrographs of MDA-MB-231 cells treated with 10-100 μg/mL doses  of PDSE at 24 and 48 h, respectively. Scale bar = 100 μm. **c** Dose-response effect of PDSE at various concentrations on percent cell viability of MDA-MB-231 cells at 24 h and 48 h using MTT assay. **d** and **e** Dose-response effect of standard compounds rutin and quercetin, respectively at various concentrations on percent cell viability of MDA-MB-231 cells at 24 h and 48 h. **f** Apoptosis-inducing activity of PDSE showing chromatin condensation in MDA-MB-231 treated cells at 50 and 100 μg/mL of PDSE at 48 h using Hoechst 33258 staining. Data are presented  as mean ± SEM of three independent experiments. .^*^p < 0.05 as compared to control

**Fig. 3:**
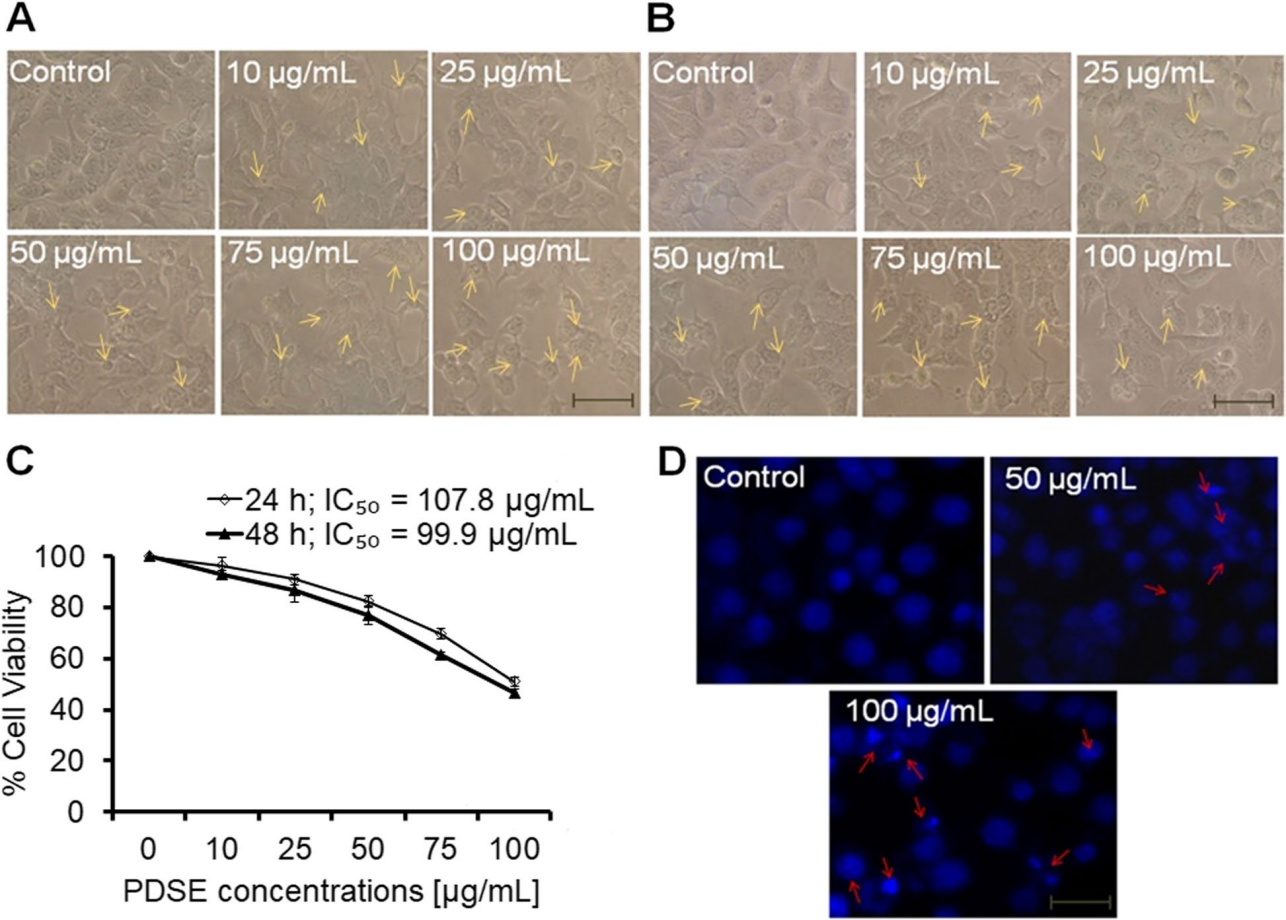
Evaluation of the cytotoxic activity of PDSE against human MCF-7 cells at different concentrations (10–100 μg/ml) using phase contrast microscope. **a** and **b** Photomicrographs of MCF-7 cells treated with 10–100 μg/mL concentrations of PDSE at 24 and 48 h, respectively. Scale bar = 100 μm. **c** Dose-response effect of PDSE at various concentrations on percent cell viability of MCF-7 cells at 24 h and 48 h using MTT assay. **d** Apoptosis-inducing activity of PDSE showing chromatin condensation in MCF-7 treated cells at 50 and 100 μg/mL of PDSE at 48 h using Hoechst 33258 staining. Values are presented  as mean ± SEM of three independent experiments. ^*^p < 0.05 as compared to control

**Fig. 4:**
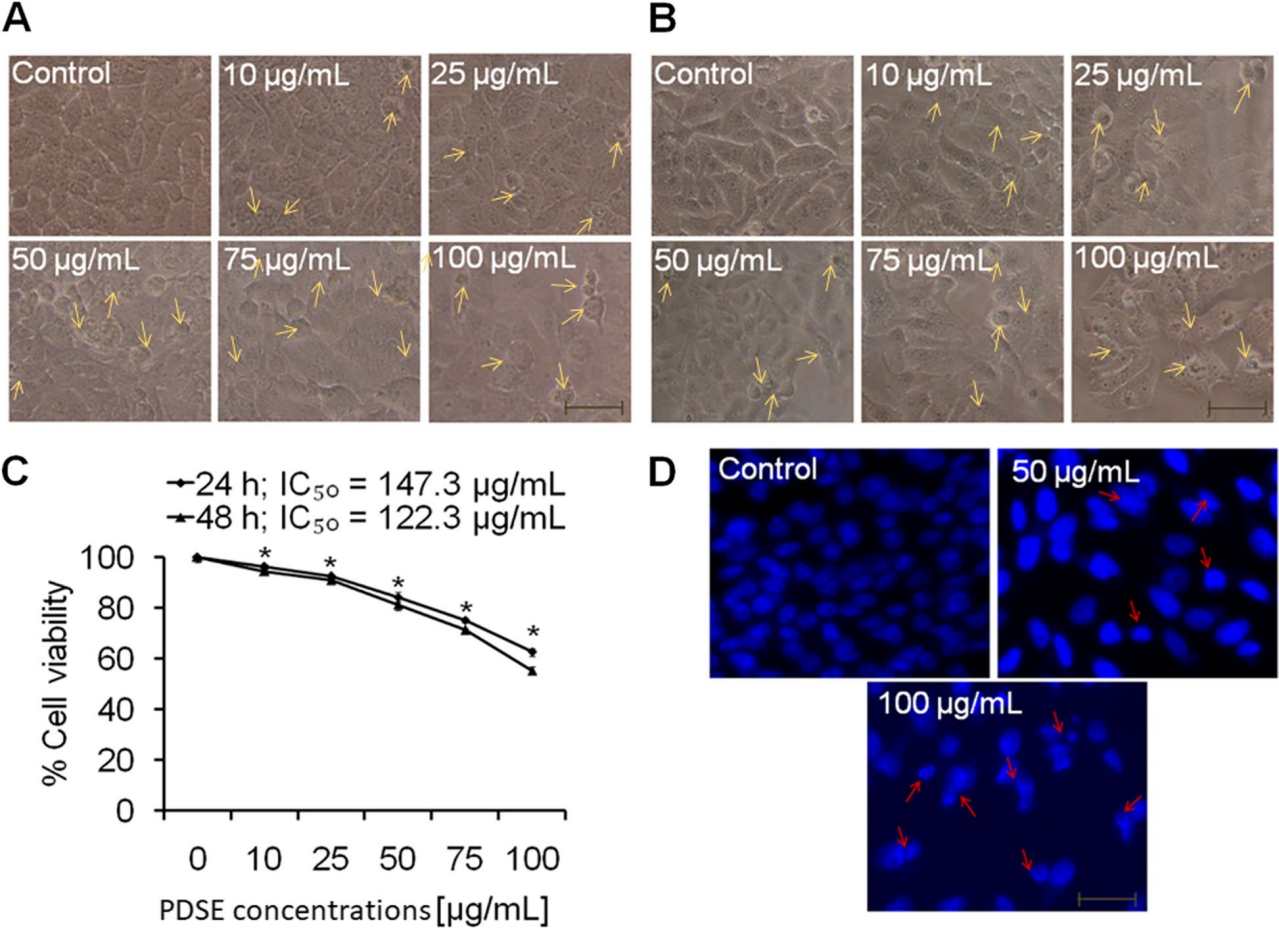
Evaluation of the cytotoxicactivity of PDSE against human HepG2 cells at different concentrations (10–100 μg/ml) using phase contrast microscope. **a** and **b** Photomicrographs of HepG2 cells treated with 10–100 μg/mL concentrations of PDSE at 24 and 48 h, respectively. Scale bar = 100 μm. **c** Dose-response effect of PDSE at various concentrations on percent cell viability of HepG2 cells at 24 h and 48 h using MTT assay. **d** Apoptosis-inducing activity of PDSE showing chromatin condensation in HepG2 treated cells at 50 and 100 μg/mL of PDSE at 48 h using Hoechst 33258 staining. Data are presented as mean ± SEM of three independent experiments. ^*^p < 0.05 as compared to control

After 24 and 48 h treatment with PDSE, MDA-MB-231, MCF-7, HepG2 and Vero cell lines were photographed . PDSE-mediated morphological variations were observed in treated and untreated cells. As shown in Figs. 2a and b; 3a and b; 4a and b, the untreated cancer cells displayed normal characteristics  *viz.*  typical adherent, uniform and even cell surface at both incubation periods. After a 24 h exposure, the majority of cancer cells formed a non-adherent, detached and rounded shape.  Whereas, after 48 h incubation period, PDSE increased the drastic morphological changes like typical apoptotic features in cancer cells. Thus, PDSE showed both dose- and time-dependent morphological effects. The MTT data of the MDA-MB-231 cell line revealed that PDSE reduced cell viability to 91.9, 87.8, 74.3, 61.7 and 48.4% at 10, 25, 50, 75 and 100 μg/mL PDSE, respectively, at 24 h. Conversely, PDSE exerted a more pronounced effect at 48 h, drastically reducing the viability of MDA-MB-231 treated cells to 90.5, 84, 67.6, 54.9 and 43.6% at 10, 25, 50, 75 and 100 μg/mL of PDSE, respectively (Fig. 2c). Figure 2d and e show the dose-response effect of standards rutin and quercetin, respectively at various concentrations on percent cell viability of MDA-MB-231 cells for 24 h and 48 h. The IC_50_ value of PDSE was found to be 101.6 and 85.86 μg/mL at 24 and 48 h, respectively. In the case of standard rutin, it was found to be 555.24 and 243.75 μM at 24 and 48 h, respectively while quercetin displayed IC_50_ values 285.52 and 169.05 μM at 24 and 48 h, respectively.

**Fig. 5:**
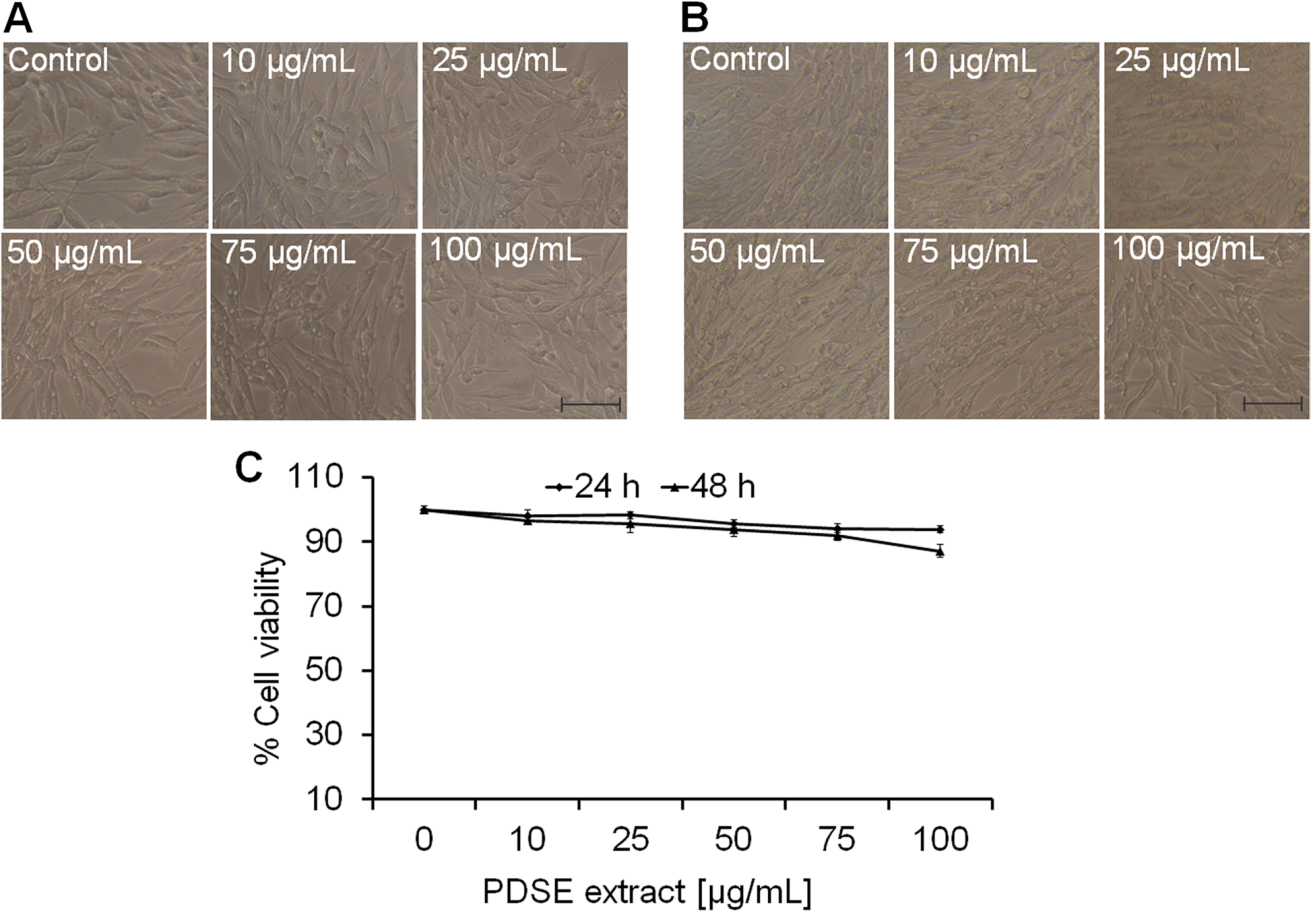
Evaluation of the cytotoxicactivity of PDSE against normal kidney epithelial cell line Vero at different concentrations (10–100 μg/ml) using phase contrast microscope. **a** and **b** Photomicrographs of Vero cells treated with 10–100 μg/mL concentrations of PDSE at 24 and 48 h, respectively. Scale bar = 100 μm. **c** Dose-response effect of PDSE at various concentrations on percent cell viability of Vero cells at 24 h and 48 h using MTT assay. Data are presented as mean ± SEM of three independent experiments

Likewise, PDSE at 10, 25, 50, 75 and 100 μg/mL decreased the cell viability to 96.4, 90.9, 82.3, 69.6 and 51% post 24 h incubation; and to 92.8, 86.5, 77, 61.3 and 46.4% at 48 h incubation, respectively in MCF-7 cell line (Fig. 3c). Similarly, PDSE at concentrations 10, 25, 50, 75 and 100 μg/mL decreased the cell viability to 96.1, 92.3, 84.3, 75.1 and 62.6% at 24 h incubation; and 94.5, 90.8, 81.1, 71.1 and 55.1% at 48 h incubation, respectively in HepG2 cell line (Fig. 4c). The cell viability data suggested that PDSE treatment significantly reduced cancer cell growth in both doses- and time-dependent manner. However, PDSE did not exert any significant morphology variation and effect on survival of normal cell line Vero as represented in Fig. 5a and b at both 24 and 48 h, respectively. The percent cell viability of Vero cells at concentrations 10, 25, 50, 75 and 100 μg/mL was found to be 98.0, 98.2, 95.5, 94.2 and 93.8% at 24 h incubation and 96.7, 95.6, 93.6, 91.9 and 87.2% at 48 h incubation period, respectively (Fig. 5c).

### Apoptotic body formation by PDSE

Based on cell viability data, two effective doses 50 and 100 μg/mL were selected to study the chromatin condensation in different cancer cell lines at 48 h incubation period. As apparent from the photomicrograph (Fig. 2f), PDSE at 50 μg/mL concentration increased the chromatin condensation in MDA-MB-231 cells as compared to control, however, 100 μg/mL of PDSE exhibited comparatively greater nuclear condensation under the inverted fluorescence microscope. Similar effects of nuclear condensation were observed against both MCF-7 and HepG2 cell lines as shown in Figs. 3d and 4d, respectively. Figures 2f, 3d and Fig. 4d show that PDSE induced pronounced nuclear apoptosis in MDA-MB-231 cell lines when compared to other cancer cells.

### PDSE causes intracellular ROS production 

**Fig. 6:**
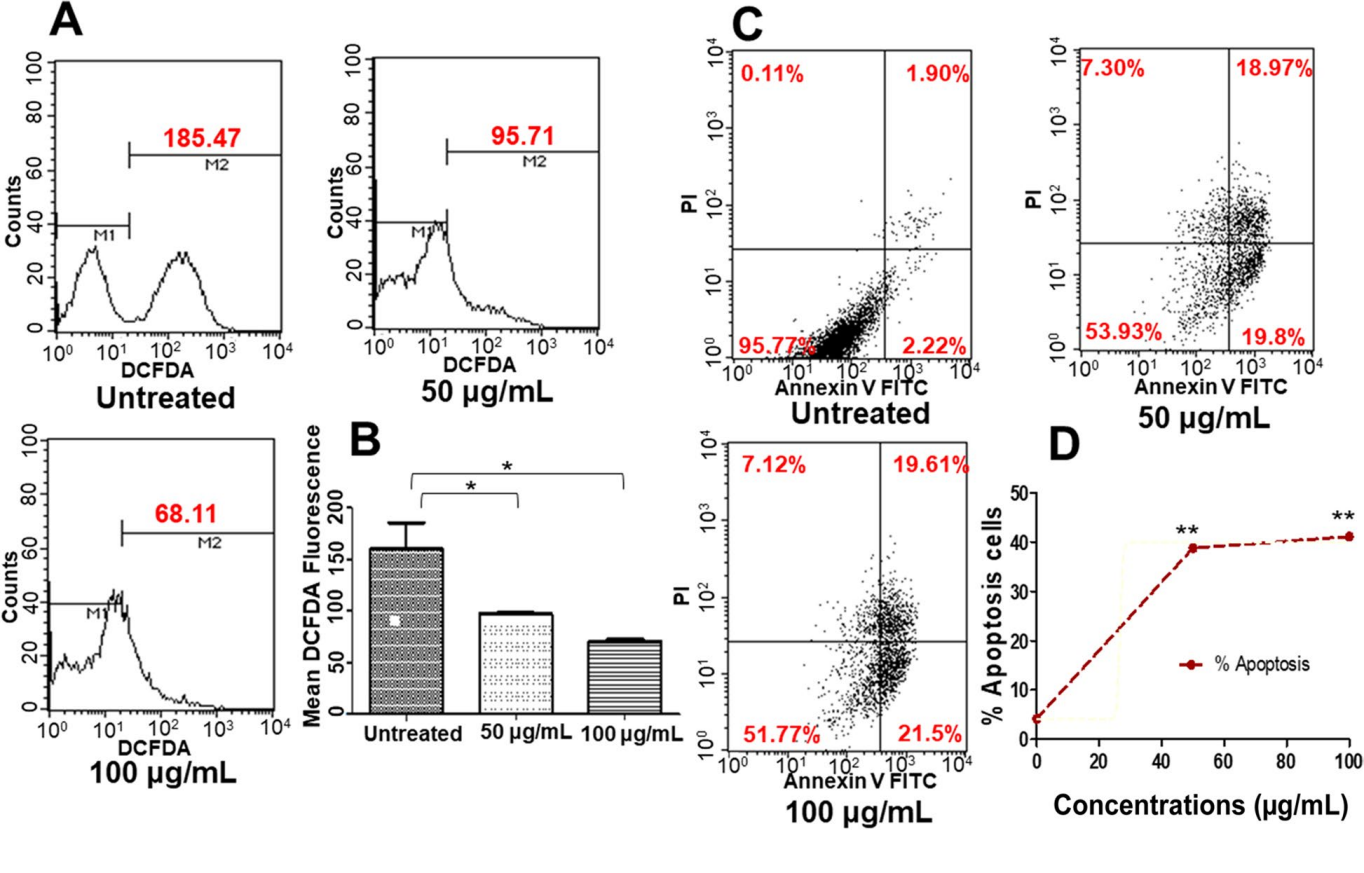
Intracellular ROS generation and apoptosis-inducing activityof PDSE in human MDA-MB-231 cells using DCFH-DA and annexin V/FITC & PI double stains, respectively. **a** DCFDA fluorescence in the cells is represented as the percentage of ROS production analyzed using flow cytometry **b** Graph showing mean fluorescence intensity. **c** Representative scatter plot from flow cytometry showing the population of viable (annexin V^−^ PI^−^), early apoptotic (annexin V^+^ PI^−^), late apoptotic (annexin V^+^ PI^+^) and necrotic (annexin V^−^ PI^+^) cells. **d** Graph showing total percentage of apoptotic cells analyzed by flow cytometry. ^*^p < 0.05 and ^**^p < 0.01 when compared to the control group

ROS measurement through flow cytometry analysis revealed that the mean fluorescence intensity of DCFDA dye in MDA-MB-231 control cells was 185.47 which was decreased to 95.71 and 68.11 at 50 and 100 μg/mL of PDSE treatment, respectively indicating a decrease in intracellular ROS levels (Fig. 6a and b).

### Apoptosis quantification at early and late stage 

Apoptosis Detection Kit (Annexin V-FITC) was used further to examine the early and late apoptotic cells. Untreated cells demonstrated negligible apoptosis and dead cells, while PDSE at 50 μg/mL promoted cell death by lowering the number of viable cells and enhancing the percentage of early (19.8%) and late (18.97%) apoptotic cells. The concentration 100 μg/mL promoted the proportion of early and late apoptotic cells to 21.5 and 19.61%, respectively (Fig. 6c and d). This indicates that a high dose of PDSE induced early and late apoptosis in treated cells.

### Decrease in MMP by PDSE

**Fig. 7:**
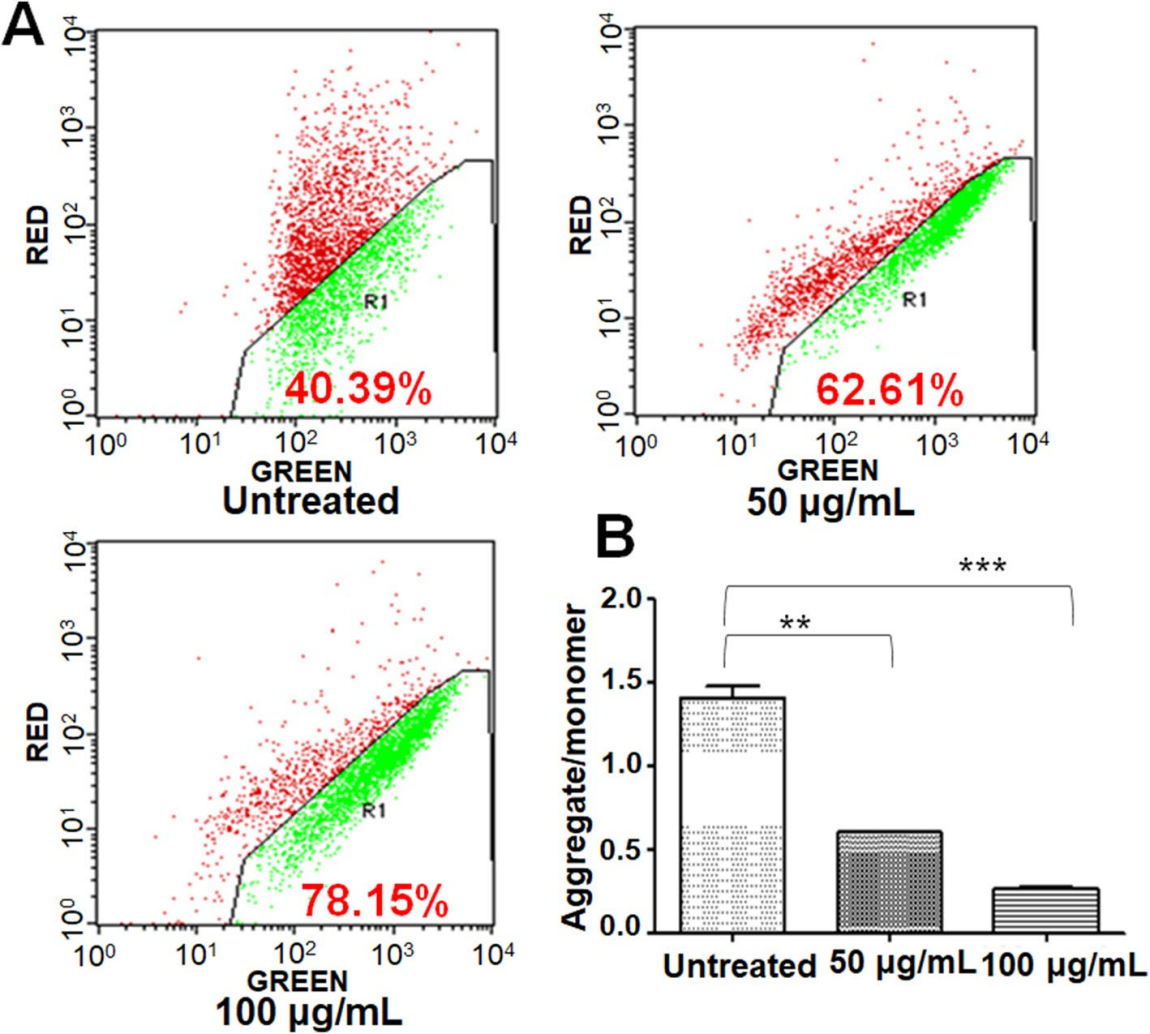
Concentration dependent effects of PDSE on the MMP changes in human MDA-MB-231 cells analyzed using JC-1 dye by flow cytometry. **a** JC-1 fluorescent dyes can aggregate in the matrix of mitochondria and produce red fluorescence. As MMP declines, JC-1 cannot aggregate so JC-1 exists in the matrix as a monomer, producing green fluorescence **b** Graph showing ratio of aggregate/monomer fluorescence in the cells. As the concentration of PDSE increased, the ratio declined. ^*^p < 0.05, ^**^p < 0.01 and ^***^p < 0.001 as compared to control group

As evident from flow cytometry data (Fig. 7), PDSE treatment resulted in an increase in green fluorescence by 62.61% at 50 μg/mL and 78.15% at 100 μg/mL doses as compared to untreated control. Further, a decrease in the ratio of aggregate (red fluorescence) to monomer (green fluorescence) was observed after PDSE treatment in MDA-MB-231 cells indicating a loss in mitochondrial membrane potential.

### Induction of S phase arrest by PDSE

**Fig. 8:**
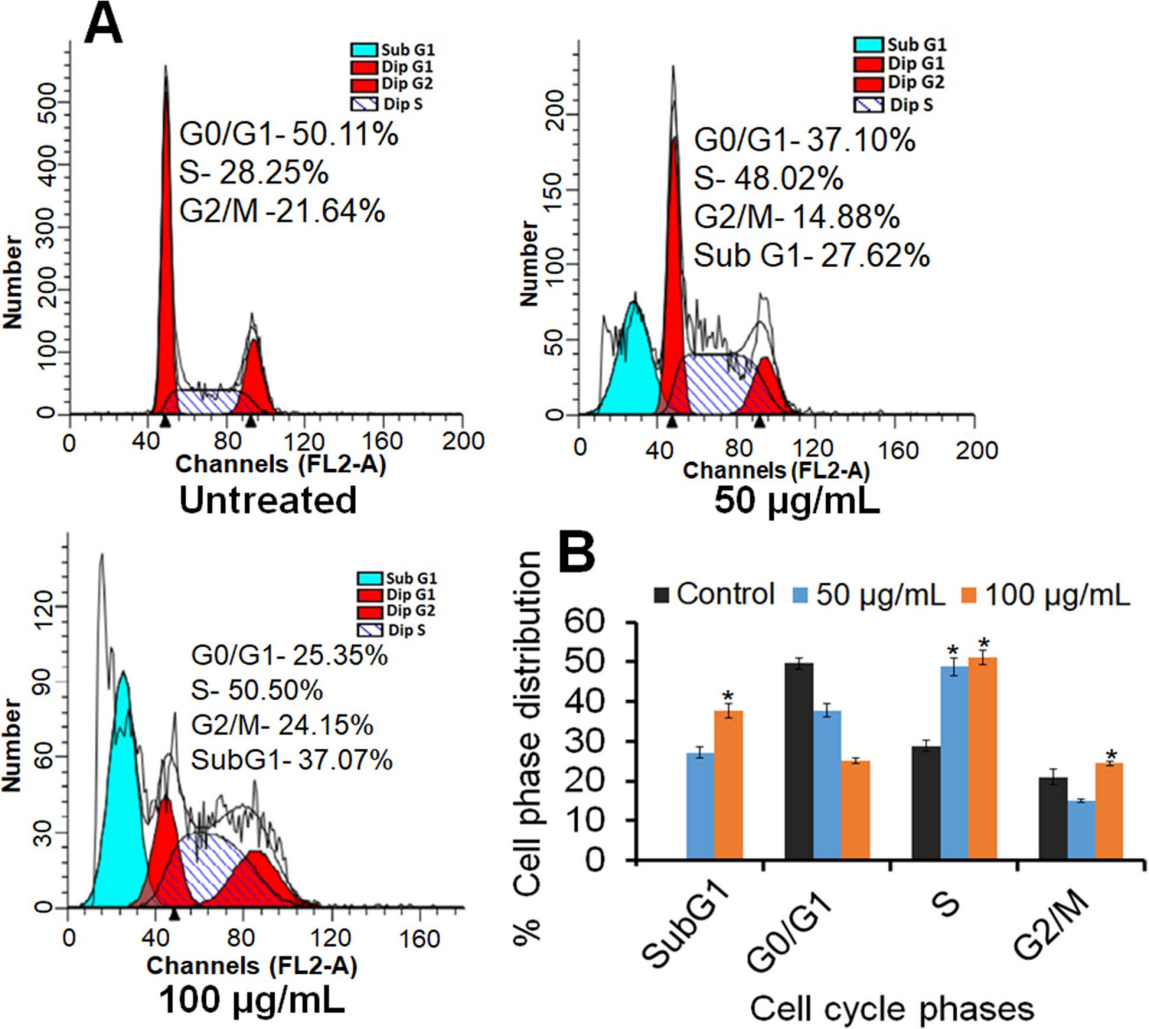
Flow cytometry results depicting effect of PDSE on cell cycle. **a** Histogram from flow cytometry showing the percentage of MDA-MB-231cells in different phases of cell cycle treated with 50 and 100 μg/mL of PDSE for 48 h **b** Quantification of flow cytometry data shown in right lower panel

Treated and untreated MDA-MB-231 cells were analyzed for cell cycle check points through flow cytometer. Figure 8a depicts the cytogram showing the proportion of MDA-MB-231 cells in different phases of the cell cycle and Fig. 8b represents the quantification of flow cytometry data. As revealed by cell cycle data (Fig. 8a), PDSE increased the DNA content (28.25% in untreated control versus 48.02% at 50 μg/mL and 50.50% at 100 μg/mL of PDSE) in the S phase of MDA-MB-231 cells with a concomitant decrease in the percentage of cells in the G0/G1 phase (50.11% in untreated control versus 37.10% at 50 μg/mL and 25.35% at 100 μg/mL of PDSE). PDSE also increased the percentage of SubG1 cells viz. 27.62 and 37.07% at 50 and 100 μg/mL as compared to untreated control. These results indicate that PDSE arrested the MDA-MB-231 cells in the S phase of the cell cycle.

### Effects of PDSE on anti-apoptotic and pro-apoptotic proteins 

**Fig. 9:**
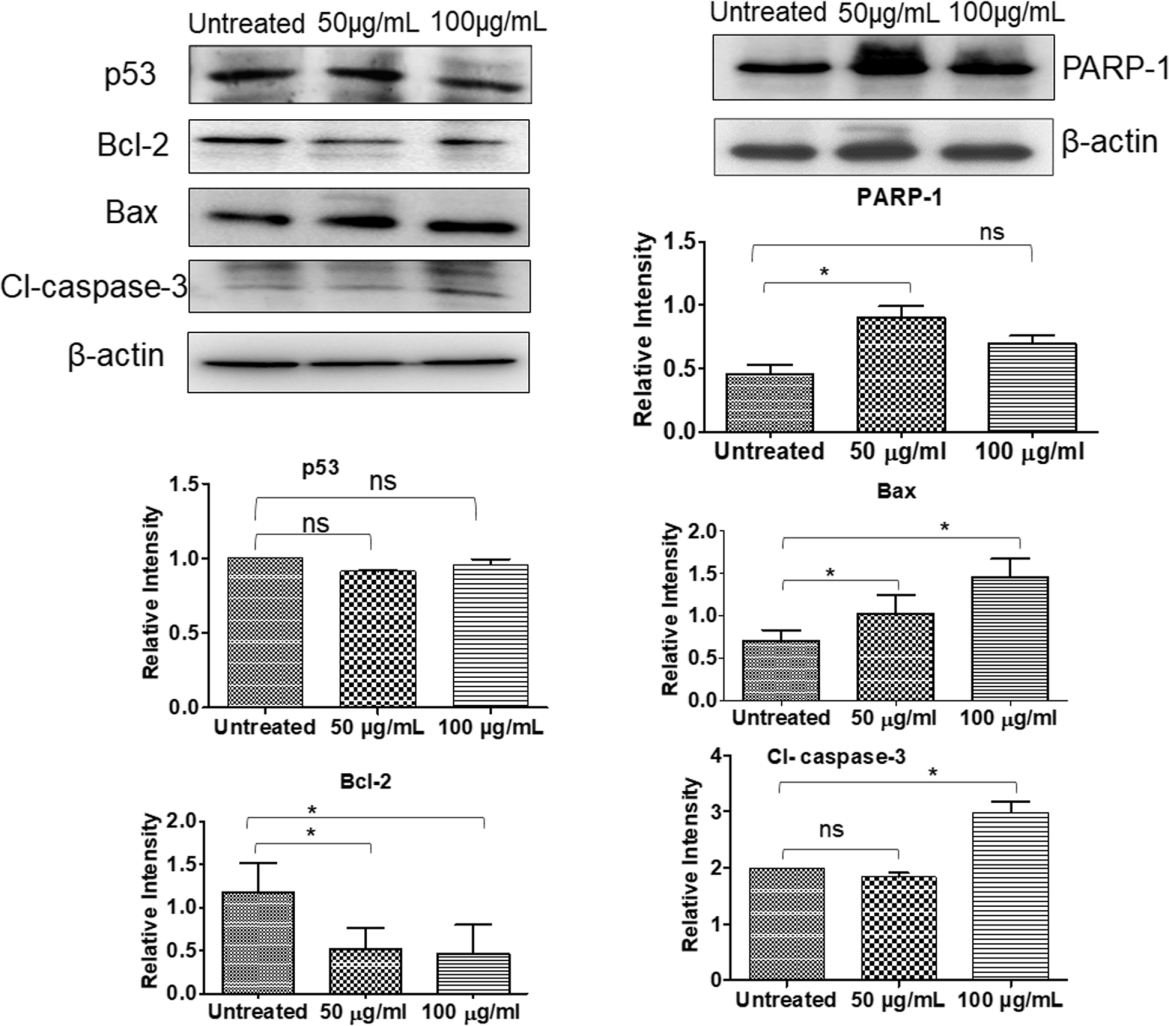
Immunoblot analysis showing the expression levels of p53, Bax, Bcl_2_, cleaved Caspase-3 and PARP-1 cleavage. MDA-MB-231 cells were treated at two effective concentrations of PDSE (50 and 100 μg/mL) for 48 h. Equal amounts of protein samples (30 μg/lane) were resolved on SDS-PAGE gel and transferred to nitrocellulose membrane. Expression of p53, Bax, Bcl_2_, cleaved Caspase-3, PARP-1 cleavage and β-actin were detected using specific antibodies. Lane 1: 0 μg/mL (Untreated); Lane 2: 50 μg/mL; Lane 3: 100 μg/mL of PDSE. Protein markers p53, Bcl2, cleaved Caspase-3, and one of the β-actin proteins were cropped from different parts of the same blot, while Bax, PARP-1 cleavage, and other β-actin proteins were cropped from different blots. β-actin was used as a loading control. The full-length blots were cut prior to antibody hybridization and each section was incubated with primary antibody individually. A high background signal was seen in the p53 blot, masking the expression of p53, thus this blot was excised at the time of developing the film. Full-length blots are shown in Supplementary Fig. S1. The data represents the mean ± SEM of three independent experiments. ^*^p < 0.05 compared to the control group

To analyze the underlying mechanisms of PDSE-induced cell death, western blotting was performed for the expression analysis of key apoptotic proteins *viz.* pro-apoptotic Bax, tumor suppressor p53, anti-apoptotic Bcl-2, effector caspase-3 and cleaved PARP-1. Results showed that pro-apoptotic protein Bax was upregulated, but it was independent of tumor suppressor protein p53 while anti-apoptotic protein Bcl-2 was down-regulated in 100 μg/mL PDSE treated cells. The cleaved caspase-3 protein expression was augmented in the MDA-MB-231 cancer cells, after 48 h of PDSE treatment. The expression of p53 did not increase significantly which means that PDSE induced cell death via a p53 independent pathway. PARP1, an enzyme involved in the repair of damaged DNA, is a preferential substrate for caspase-3. PDSE caused the cleavage of PARP1 in a dose-dependent manner in MDA-MB-231 cells (Fig. 9). These results indicate that PDSE induces apoptosis through intrinsic apoptotic pathways in breast cancer cells.

###  Binding analysis of rutin and quercetin present in  PDSE against caspase-3 protein

**Fig. 10:**
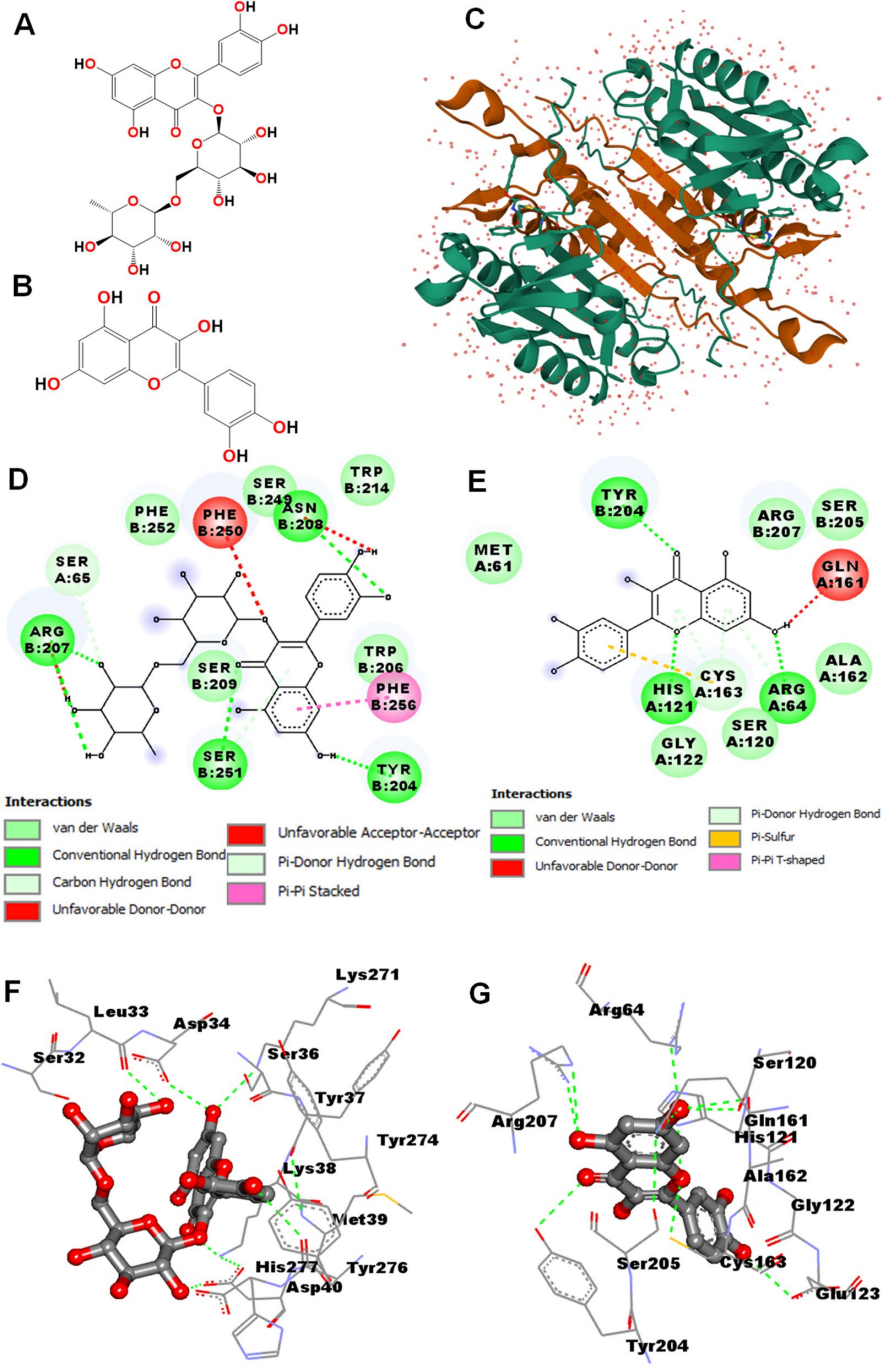
Visualization of binding poses of rutin and quercetin in the binding site of caspase-3 protein (PDB ID: 2XYP; Hetero 4-mer - A2B2) using Accelrys Biovia Discovery Studio 2017 R2. **a** Molecular structure of rutin **b** Molecular structure of quercetin **c** 3-D crystal structure of Caspase-3 protein **d** & **e** Docking interaction of rutin-caspase-3 complex and quercetin-caspase-3 complex analyzed by AutoDock Vina, respectively. **f** & **g** Docking interaction of rutin-caspase-3 complex and quercetin-caspase-3 complex analyzed by iGEMDOCK v2.1, respectively. In AutoDock Vina, the ligand-protein complex is represented by the 2-D line model, whereas in iGEMDOCK v2.1 analyses, the ligand is represented by the stick model. The dark green dotted line represents H - bond

The docking tools AutoDock Vina and iGEMDOCK v2.1 were used to study the binding interaction of rutin and quercetin present in PDSE with a caspase-3 target protein. The docking results obtained from both docking tools were visualized by BIOVIA Discovery Studio software. AutoDock Vina is based on the statistical scoring function that replaces the semi-empirical free energy force field of AutoDock 4.2. AutoDock Vina provides improved prediction accuracy and speed, which is not only due to the simplification of the scoring function but also due to the capability of multi-threading in presence of multiple CPU cores. Figure 10a and b depict the molecular structure of rutin and quercetin, respectively; Fig. 10c represents the 3-D crystal structure of a caspase-3 protein; Figs. 10d and Fig. 10e depict the docking interaction of rutin and quercetin complexed with caspase-3 protein, respectively as analyzed by AutoDock Vina while Fig. 10f and g are destined for rutin and quercetin, respectively as analyzed by iGEMDOCK v2.1 tool. Table 1 represents the active constituents of PDSE with their chemical structure, binding energy, dissociation constant, best docking poses with amino acid residues contributing to the binding pocket of the caspase-3 protein. As shown in Table 1, rutin and quercetin phytoconstituents exhibited potent binding interaction with caspase-3 protein. As analyzed by AutoDock Vina, the binding energy of rutin (BE = − 9.1 kcal/mol) was found to be lower than the binding energy of quercetin (BE = − 7.6 kcal/mol), which means that the binding affinity of rutin with caspase-3 protein is greater than quercetin. These results were further confirmed from analysis using iGEMDOCK v2.1, which showed consistent results with both components. The result suggests that both ligands have different binding sites and hence possess different binding energies towards caspase-3 protein.

**Table 1:** Docking interactions of rutin and quercetin active components present in PDSE with executioner caspase-3 protein (PDB ID: 2XYP; Hetero 4-mer - A2B2) using AutoDock Vina and iGEMDOCK v2.1

	AutoDock Vina				iGEMDOCK v2.1			
S. No.	Ligands with MF, and MW	BE (kcal/mol)	Interacting amino acids	T.E. (kcal/mol)	VDW	HB	EI	Interacting amino acids
1.	Rutin PubChem CID: 5280805 MF: C_27_H_30_O_16_ MW: 610.5 g/mol Chemical Class: Flavonol Glycoside	−9.1	Ser65, Arg207, Ser209, Phe250, Asn208, Trp214, Ser249, Trp206, Phe256, Tyr204, ser251, Phe252	−103.31	−78.02	−25.29	0	Leu33, Ser32, Asp34, Ser36, Lys271, Tyr37, Tyr274, Lys38, Met39, Tyr276, His277, Asp40
2.	Quercetin PubChem CID: 5280343 MF: C_15_H_10_O_7_ MW: 302.23 g/mol Chemical Class: Polyphenolic Flavonoid	−7.6	Met61, Tyr204, Arg207, Ser205, Gln161, Ala162, Arg64, Ser120, **Cys163, His121**, Gly122	−113.04	−82.16	−30.88	0	Arg64, Ser120, Gln161, **His121**, Ala162, Gly122, Glu123, **Cys163**, Ser205, Tyr204, Arg207

Note: Bold letters display the catalytic residues in caspase-3 protein

## Discussion

Cancer is a foremost health problem that affects millions of people across the world. At present, chemotherapy is considered one of the most effective cancer treatment strategies. Although chemotherapy significantly improves the prognosis of cancer patients, but this approach is not free from side effects. Chemotherapeutic drugs can damage normal, healthy cells resulting in side effects like hair loss, anemia, sores, nerve, muscle as well as kidney and fertility problems [29]. To avoid side effects, most cancer patients adopt alternative therapy based on herbal medicines. Plant-based cancer treatment is considered a better treatment option because phytotherapeutic agents are natural, readily available, easily assimilated in the body, and have fewer side effects and toxicity.

**Fig. 11:**
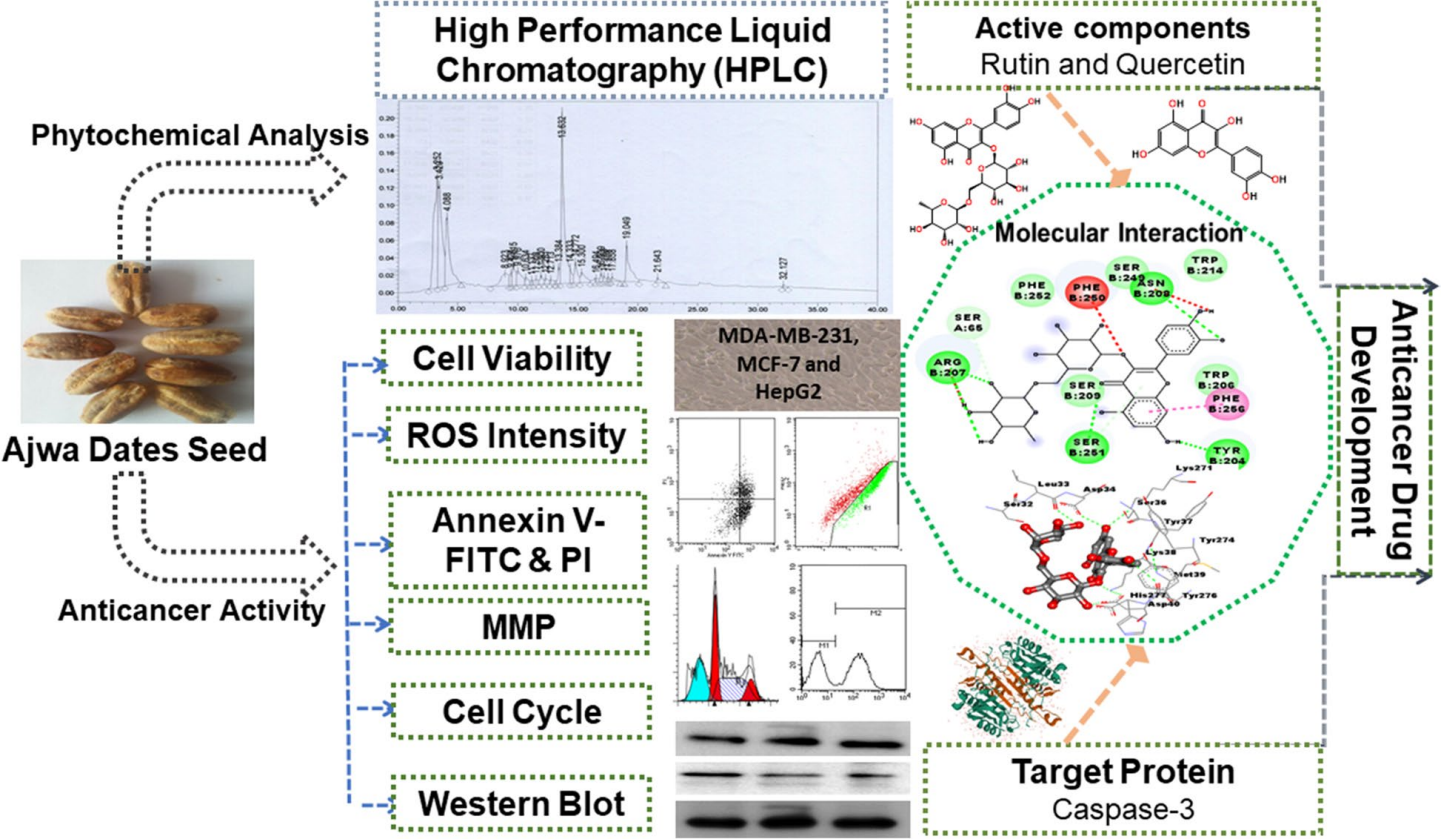
A comprehensive summary of the current study representing the phytochemical analysis of PDSE, in vitro anticancer study against liver and breast cancer cells and in silico analysis of PDSE active components with caspase-3 targeted protein

Medicinal plants are well known for their antioxidant and immunomodulatory properties, as well as their anticancer activities [9, 30]. The seeds of Ajwa dates are abundant in minerals, vitamins, dietary fibers, phenolic compounds, and different flavonoids [17]. Evidence indicates that individuals with a high intake of dietary fiber and phenolic compounds have reduced incidence of colorectal, prostate, lung, breast and ovarian cancer(s) [31–33]. Previous studies have shown that date palm seeds induce immunity in broiler chickens [34], cause an increase in the paraoxonase and arylesterase activities in hypercholesterolemic rats [35] and enhance the endogenous insulin secretion in type 1 diabetic rats [36]. An earlier study has also found that a combination of black pepper and Ajwa seed extract normalizes glucose levels and liver enzymes aspartate transaminase, alanine transaminase and alkaline phosphatase activities in alloxan-induced diabetic rats [37]. The therapeutic potential of PDSE has been investigated against DNA damage induced by N-nitroso-N-methyl urea (NMU) in mice [38] and carbon tetrachloride (CCl4)-induced hepatotoxicity in rats [15]. The protective role of PDSE against gastric ulcers has also been investigated [39]. Two ex vivo studies have been carried out to evaluate the efficacy of date seed oil extract to prevent oxidative stress; a major contributor towards cancer development [40, 41]. Moreover, a study has shown that acetone extract of date palm seeds is highly cytotoxic against human colorectal cancer cell line HCT-15 and has significant antibacterial activity against *Bacillus cereus* and *Escherichia coli* [42]. However, further experimental work needs to be performed to confirm the previous traditional applications of Ajwa date seeds in cancer treatment. The current study not only presents a novel approach to understand the anticancer activity but also augments the existing knowledge about the traditional use of the PDSE. Although previous studies have reported the antidiabetic, hypolipidemic and antioxidant properties of PDSE [43, 44], the cytotoxic effects of PDSE against human breast cancer cell lines MDA-MB-231 and MCF-7 and human liver cancer cell line HepG2 remain to be investigated. This study also attempted the HPLC characterization of PDSE to find the major active component(s) that might be contributing to the anticancer potential of the extract. Moreover, in silico molecular docking analysis between active components, viz. rutin and quercetin with apoptosis executioner caspase-3 protein further validated the anticancer potential of PDSE. For a comprehensive summary, Fig. 11 summarizes the phytochemical analysis, several in vitro anticancer parameters, and in silico analysis.

The cell viability data indicated that PDSE had a cytotoxic effect against MDA-MB-231 cells with IC_50_ values of 101.6 and 85.86 μg/mL following 24 and 48 h incubation, respectively. Likewise, PDSE induced cell death in MCF-7 cell line having IC_50_ values of 107.8 and 99.9 μg/mL following 24 and 48 h incubation, respectively; and IC_50_values of 147.3 and 122.3 μg/mL against HepG2 cell line following 24 and 48 h incubation, respectively. PDSE toxicity was shown to be higher in MDA-MB-231 cells (IC_50_ = 85.86 μg/mL) than in the other two cancer cell lines (MCF-7 and HepG2 cells). PDSE showed modest cytotoxicity on each kind of cancer cell after 48 h, resulting in minor changes in IC_50_ value. Cytotoxic potential of PDSE was reduced in the following order: MDA-MB-231 > MCF-7 > HepG2. MDA-MB-231 cells exhibit an estrogen-independent state and do not express estrogen receptors and hence they are ideal models for chemotherapeutic studies, however, MCF-7 cells possess estrogen and progesterone receptors and hence they are suitable models for investigations on hormone therapy [45]. Based on this principle, it can be concluded that PDSE could be a better therapeutic agent for the growth inhibition of triple-negative breast cancer cell line MDA-MB-231. A previous study has reported that hydromethanolic extract of *Ardisia crispa* showed moderate cytotoxic effect against MCF-7 and a weak cytotoxic effect against MDA-MB-231 [46]. Conversely, another study has stated that hexane fraction of *Acanthopanax sessiliflorus* stem bark extract displayed more cytotoxicity against MDA-MB-231 cells compared to MCF-7 cells [47]. Interestingly, in agreement with this previous study, ethanolic PDSE was found to be more cytotoxic against MDA-MB-231 cells as compared to MCF-7 cells. In addition, PDSE did not exert any significant morphology variation and toxic effect on survival of normal cell line Vero as represented (Fig. 5). Furthermore, the present study involved an investigation into the mechanism(s) responsible for PDSE mediated cytotoxicity. There are two major pathways of cell death viz. apoptosis or necrosis. Results of nuclear condensation by Hoechst staining revealed that PDSE-treated cancer cells exhibited the hallmark features of apoptosis viz. cell shrinkage, nuclear chromatin condensation and formation of fragmented apoptotic bodies [23] (Figs. 2f, 3d, and 4d). Previous studies have also reported the apoptotic effect of several plant extracts against different cancer cell lines [48–50].

In cancer cells, high levels of ROS have been detected due to increased metabolic and peroxisomal activities, mitochondrial dysfunction, increased receptor signaling, oncogenic activity, increased enzymatic activity of oxidases, lipoxygenases, cyclooxygenases and thymidine phosphorylase [51]. In this study, PDSE decreased the level of ROS in treated MDA-MB-231 cells, which indicates that PDSE induced cell death independent of ROS pathway. ROS-independent cell apoptosis pathway has rarely been reported in cancer cells and may act as a natural regulator of important signaling pathways in cells [52]. Therefore, it can be expected that ROS signaling does not influence the mechanisms of PDSE-induced cell death. Interestingly, in earlier research, several prospective compounds viz. metformin, quercetin, curcumin and vitamin C have been found to downregulate ROS in the cellular apoptotic process and some of them have even been demonstrated to promote apoptosis in cancer cells [53]. Further, based on initial study of nuclear condensation, MDA-MB-231 cells were stained with Annexin-V-FITC/PI double stain and examined by flow cytometry. Results showed that the percentage of live cells was decreased with a simultaneous increase in early and late apoptosis of the cells. This study suggests that high dose of PDSE increases the percentage of cells undergoing late apoptosis (Fig. 6c). Similarly, in a previous study, methanolic extract of Ajwa dates pulp has been reported to increase the percentage of MCF-7 cells undergoing late apoptosis [54].

Mitochondria is not only the ATP factory of energy but also help in the regulation of the membrane potential, apoptosis, calcium signaling and regulation of cellular metabolism [55]. The efficacy of cancer therapy can be improved by altering the cellular metabolism of tumor cells or promoting MMP decrease. Permeabilization of the inner mitochondrial membrane causes disruption of MMP and thus, a decline of MMP is associated with the opening of a mitochondrial permeability transition pore which results in the rupture of the outer mitochondrial membrane and release of various apoptosis factors such as Cyt c, Smac, Endo G into the cytoplasm, finally leading to cell apoptosis [56]. The present study revealed that PDSE declined the MMP level by changing the JC-1 fluorescent color from red to green, suggesting that depletion of MMP is associated with the increasing concentration of PDSE. Cell cycle analysis showed that PDSE treatment resulted in S phase arrest of cell cycle followed by apoptosis in MDA-MB-231 cancer cells (Fig. 5). This study confirmed that PDSE hampers the initiation of DNA replication and thus arrests MDA-MB-231 cells at the S phase. Apoptotic events also require permeabilization of the outer membrane of the mitochondria which is regulated by the Bcl-2 family proteins. Based on the role of Bcl-2 family proteins, this study was also formulated to analyze protein expression of p53, Bax, Bcl-2, cleaved caspase-3 and PARP-1 cleavage in PDSE- treated cancer cells. Western blot analysis revealed that PDSE increased the expression level of pro-apoptotic Bax and effector cleaved caspase-3, whereas PDSE downregulated the anti-apoptotic Bcl-2 protein level (Fig. 9). While, PDSE did not reduce the tumor suppressor p53 protein, indicating PDSE induced cell death via p53 independent pathway. Following activation, Bax and Bak form homo-oligomers deactivating Bcl-2 protein and contribute to pore formation which causes permeabilization of the outer mitochondrial membrane, leading to the release of mitochondrial inner membrane space contents, including Cyt c and Smac, into the cytosol [57]. These contents drive the activation of apoptotic effector caspases including caspase-3 that cleave and degrade the crucial PARP protein, a DNA repair enzyme leading to DNA breakage and cellular apoptosis [58]. The apoptosis executioner enzyme caspase-3 is the main proteolytic cascade involved in the apoptosis of both intrinsic and death receptor pathways [59]. Western blot data indicated that PDSE induced cellular apoptosis through intrinsic pathways in breast cancer cells.

PDSE was further characterized using HPLC to identify the phytoconstituents present therein. Rutin and quercetin were the principal phytochemicals identified in PDSE. Rutin and quercetin are the most widely distributed plant flavonoids and major constituents of the human diet [60, 61]. Previous studies have confirmed the anticancer activities of both bioactive agents against different human cancer cell lines [62–64]. In our recent publications, the pulp extract of Ajwa dates has shown IC_50_ values of 20.03 and 16.78 mg/mL at 24 and 48 h periods, respectively, against human liver cancer HepG2 cells and IC_50_ values of 17.45 and 16.67 mg/mL at 24 and 48 h, respectively against TNBC MDA-MB-231 cells [23, 65]. While the active components of Ajwa date pulp extract in the previous study was β-D-glucan (a polysaccharide), rutin and quercetin were the active phytoconstituents identified in PDSE. Lower doses of PDSE were required as compared to Ajwa dates pulp extract, which suggests that seed extract has comparatively more potential against cancer cells as compared to pulp extract of Ajwa dates. Furthermore, an in silico computational study was carried out using chemoinformatic tools to validate caspase-3 activation. As caspase-3 is the main executioner protein accountable for apoptosis and also shares many structural characteristics with other-known caspases [59], thus, caspase-3 was selected as a potential target protein for ligand-protein binding affinity. Moreover, caspase-3 is active over a broad pH range [66], which indicates that caspase-3 would be fully active under normal and apoptotic cell conditions.

Caspase-3, (also called apopain) is synthesized in the cell in its zymogen form of 32 kDa, consisting of an N-terminal pro-domain followed by a large 17 kDa (p17) and small 12 kDa (p12) subunit linked to each other by an inter-subunit linker. Caspase-3 in its functional form is a heterotetramer; formed by hydrophobic interactions of four anti-parallel beta-sheets from p17 and two from p12 subunits. Beta-sheet interacts with another heterodimer resulting in a 12-stranded beta-sheet structure, around which alpha- helices are positioned. A previous study has shown that catalytic residues of caspase-3 consist of sulfhydryl group of Cys-163 and the imidazole ring of His-121 [67]. The large subunit p17 harbors the active site catalytic dyad residues and the small subunits contain most of the dimer interface and the allosteric site [68]. Interestingly, as revealed by in silico binding interaction data using AutoDock Vina and iGEMDOCK v2.1 tools, rutin present in PDSE did not interacted with any catalytic residue, while quercetin interacted with both catalytic residues His121 and Cys163 in addition to other amino acid residues in the binding pocket of caspase-3 (Table 1). As is apparent from binding interactions of amino acid residues in both AutoDock Vina and iGEMDOCK v2.1 analyses, the slight variations in interacting amino acid residues are because of the differences in the grid box generation and determination of binding pockets on the target protein [28].

## Conclusion

The current study revealed the cytotoxic activity of PDSE against breast and liver cancer and apoptotic potential against TNBC MDA-MB-231 cells. This activity could be attributed to the synergistic effect of bioactive agents particularly rutin and quercetin present in PDSE. Moreover, in silico analysis confirmed the potential binding affinity of rutin and quercetin with amino acid residues of caspase-3 executioner protein. However, because caspase-3 is a downstream protein, the apoptotic effect of PDSE in upstream pathways cannot be rationalized by binding these bioactive compounds to caspase-3. As a result, further study is warranted for upstream target-protein(s) docking studies, as well as the isolation of bioactive compounds from PDSE and their mechanisms of action in vitro and in vivo. These studies would lead to a more complete assessment of PDSE and its feasibility as a future anticancer drug candidate and an adjunct.

## Supplementary Information

**Additional file 1: Supplementary Figure S1.** Full-length blots from different gels showing the expression levels of p53, Bax, Bcl2, cleaved Caspase-3, and PARP-1 cleavage. Proteins p53, Bcl2, cleaved Caspase-3 and one of the β-actin proteins were cropped from different parts of the same blot, while Bax, PARP-1 cleavage and other β-actin proteins were cropped from different blots.

**Additional file 2: Table S1.** PASS analysis. **Table S2.** Drug-like character. **Table S3.** Admet SAR prediction. **Table S4.** Bioactivity scores. **Table S5.** Toxicity Risk Assessment.

## Data Availability

The datasets used and analyzed in the current study are included in this article.

## Abbreviations

CCl_4_: Carbon tetrachloride
DMSO: Dimethyl sulfoxide
DMEM/F-12: Dulbecco’s Modified Eagle Medium Nutrient Mixture F-12
FBS: Fetal bovine serum
HPLC: High performance liquid chromatography
MMP: mitochondrial membrane potential
MTT: 3-(4,5-dimethylthiazol-2-yl)-2,5-diphenyl-2H-tetrazolium bromide
NMU: N-nitroso-N-methylurea
PDSE: *Phoenix dactylifera* seed extract
PBS: Phosphate- buffered saline
ROS: Reactive oxygen species
RIPA: Radio immune precipitation assay
SDS-PAGE: Sodium dodecyl sulphate- polyacrylamide gel electrophoresis
TNBC: Triple negative breast cancer
