# Supplementary material for: Anticancer potential of Phoenix dactylifera L. seed extract in human cancer cells and pro-apoptotic effects mediated through caspase-3 dependent pathway in human breast cancer MDA-MB-231 cells: an in vitro and in silico investigation
Source: BMC Complement Med Ther. 2022 Mar 15;22:68. doi: 10.1186/s12906-022-03533-0 (PMC8922853; doi:10.1186/s12906-022-03533-0)
Supplement: Supplementary file 1 — Additional file 1: Supplementary Figure S1. Full-length blots from different gels showing the expression levels of p53, Bax, Bcl2, cleaved Caspase-3, and PARP-1 cleavage. Proteins p53, Bcl2, cleaved Caspase-3 and one of the β-actin proteins were cropped from different parts of the same blot, while Bax, PARP-1 cleavage and other β-actin proteins were cropped from different blots. [file 12906_2022_3533_MOESM1_ESM.pdf]

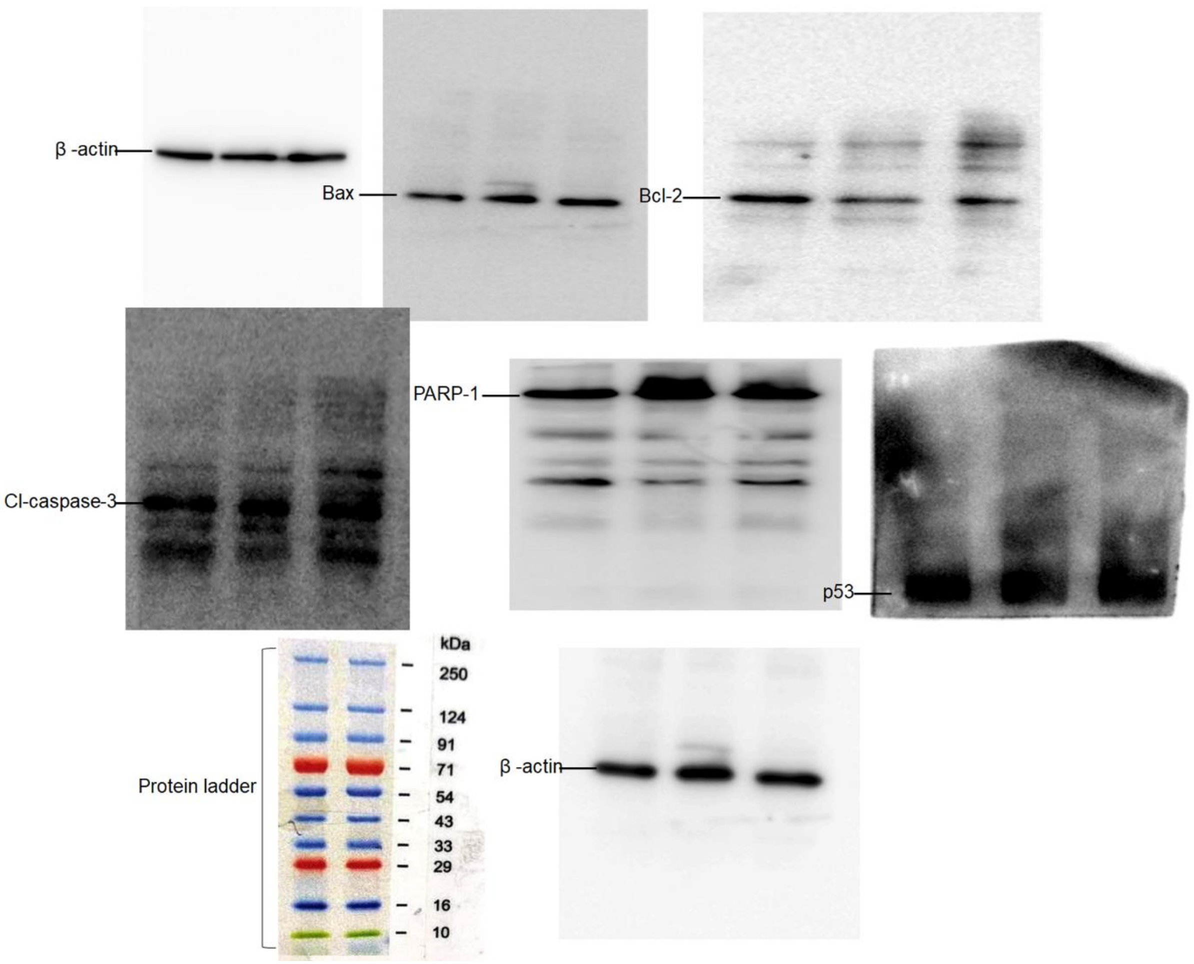

Supplementary Figure S1. Full-length blots from different gels showing the expression levels of p53, Bax, Bcl2, cleaved Caspase-3, and PARP-1 cleavage. Proteins p53, Bcl2, cleaved Caspase-3 and one of the β-actin proteins were cropped from different parts of the same blot, while Bax, PARP-1 cleavage and other β-actin proteins were cropped from different blots.
