# Supplementary material for: Anticancer potential of Phoenix dactylifera L. seed extract in human cancer cells and pro-apoptotic effects mediated through caspase-3 dependent pathway in human breast cancer MDA-MB-231 cells: an in vitro and in silico investigation
Source: BMC Complement Med Ther. 2022 Mar 15;22:68. doi: 10.1186/s12906-022-03533-0 (PMC8922853; doi:10.1186/s12906-022-03533-0)
Supplement: Supplementary file 2 — Additional file 2: Table S1. PASS analysis. Table S2. Drug-like character. Table S3. Admet SAR prediction. Table S4. Bioactivity scores. Table S5. Toxicity Risk Assessment. [file 12906_2022_3533_MOESM2_ESM.pdf]

Table S1. PASS analysis

S.No.	Ligands	Physicochemical Properties: Lipinski's rule of 5 parameters							
		% Absorption (>50%)	Topological Polar Surface Area $(Å)^2$ (TPSA)[b] (<160 Å)	MW (<500)	clog P[c] (<5)	Hydrogen Bond Donors (NOHNH) (≤5)	Hydrogen Bond Acceptors (NON) ≤10)	Number of Rotatable Bonds (≤10)	Lipinski's Violation (LV)
**1.**	**Rutin**	16.05	269.43	610.52	-1.2573	10	16	6	3
**2.**	**Quercetin**	63.68	131.35	302.24	1.4902	5	7	1	0

RULE:
[a]**Percentage Absorption was calculated as: % Absorption =109- [0.345xTopological Polar Surface Area]**
[b]**Topological polar surface area (defined as a sum of surfaces of polar atoms in a molecule).**
[c]**Logarithm of compound partition coefficient between n-octanol and water**

Table S2. Drug-like character

S.No.	Ligands	[a]G. No. of violation	[b]V. No. of Violation	[c]E. No. of violation	[d]M. No. of violation	Druglikeness
**1.**	**Rutin**	No; 4	No; 1	No; 1	No; 4	1.93
**2.**	**Quercetin**	Yes; 0	Yes; 0	Yes; 0	Yes; 0	-0.08

RULE:
[a]**Ghose filter**
[b]**Veber filter**
[c]**Egan (Pharmacial) filter**
[d]**Muegge (Bayer) filter**

Table S3. Admet SAR prediction

Ligands	Human intestinal absorption (HIA)		Caco-2 permeability		P-glycoprotein inhibitor		Blood-brain barrier penetration (BBB)		Ames mutagenesis		Subcellular localization		Biodegradation		Acute Oral Toxicity (kg/mol)
	+/-	p-value	+/-	p-value	+/-	p-value	+/-	p-value	+/-	p-value	+/-	p-value	+/-	p-value	
**Rutin**	+	0.7322	-	0.9269	-	0.6165	-	0.9393	+	0.8000	Mitochondria	0.7477	-	0.7750	2.593
**Quercetin**	+	0.9833	-	0.6417	-	0.9191	-	0.4632	+	0.9000	Mitochondria	0.5892	-	0.8250	2.559

Table S4. Bioactivity scores

S. No.	Ligands	Parameters of Bioactivity score					
		GPCR Ligand	Ion Channel Modulator (ICM)	Kinase Inhibitor (KI)	Nuclear Receptor Ligand (NRL)	Protease Inhibitor (PI)	Enzyme Inhibitor (EI)
**1.**	**Rutin**	-0.05	-0.52	-0.14	-0.23	-0.07	0.12
**2.**	**Quercetin**	-0.06	-0.19	0.28	0.36	-0.25	0.28

RULE:
BAS >0: Active
BAS -5.0 - 0.0: Moderately Active
BAS < -5.0: Inactive

Table S5. Toxicity Risk Assessment

S. No.	Ligands	Mutagenic	Tumorigenic	Reproductive Effective	Irritant
**1.**	**Rutin**	None	None	None	None
**2.**	**Quercetin**	High	High	None	None
